# Strengthening monitoring and evaluation (M&E) and building sustainable health information systems in resource limited countries: lessons learned from an M&E task-shifting initiative in Botswana

**DOI:** 10.1186/1471-2458-14-1032

**Published:** 2014-10-03

**Authors:** Mulamuli Mpofu, Bazghina-werq Semo, Jessica Grignon, Refeletswe Lebelonyane, Steven Ludick, Ellah Matshediso, Baraedi Sento, Jenny H Ledikwe

**Affiliations:** International Training and Education Centre for Health (I-TECH), P.O. Box AC46 ACH, Riverwalk, Gaborone, Botswana; Department of Global Health, University of Washington, Seattle, Washington USA; Botswana Ministry of Health, Gaborone, Botswana; Botswana Ministry of Local Government, Gaborone, Botswana; University of Botswana, Gaborone, Botswana

**Keywords:** Monitoring and evaluation, Data quality, Health information systems, Task-shifting

## Abstract

**Background:**

The demand for quality data and the interest in health information systems has increased due to the need for country-level progress reporting towards attainment of the United Nations Millennium Development Goals and global health initiatives. To improve monitoring and evaluation (M&E) of health programs in Botswana, 51 recent university graduates with no experience in M&E were recruited and provided with on-the-job training and mentoring to develop a new cadre of health worker: the district M&E officer. Three years after establishment of the cadre, an assessment was conducted to document achievements and lessons learnt.

**Methods:**

This qualitative assessment included in-depth interviews at the national level (n = 12) with officers from government institutions, donor agencies, and technical organizations; and six focus group discussions separately with district M&E officers, district managers, and program officers coordinating different district health programs.

**Results:**

Reported achievements of the cadre included improved health worker capacity to monitor and evaluate programs within the districts; improved data quality, management, and reporting; increased use of health data for disease surveillance, operational research, and planning purposes; and increased availability of time for nurses and other health workers to concentrate on core clinical duties. Lessons learnt from the assessment included: the importance of clarifying roles for newly established cadres, aligning resources and equipment to expectations, importance of stakeholder collaboration in implementation of sustainable programs, and ensuring retention of new cadres.

**Conclusion:**

The development of a dedicated M&E cadre at the district level contributed positively to health information systems in Botswana by helping build M&E capacity and improving data quality, management, and data use. This assessment has shown that such cadres can be developed sustainably if the initiative is country-led, focusing on recruitment and capacity-development of local counterparts, with a clear government retention plan.

## Background

Quality data within the health sector is crucial for monitoring and evaluation (M&E) of health program performance and for developing appropriate policies, plans, and strategies to ensure sound patient care [1]. Data quality is defined as “the totality of features and characteristics of a data set that bear on its ability to satisfy the needs that result from the intended use of the data” [2]. It includes the following dimensions: accuracy, reliability, completeness, timeliness, precision, and integrity [3]. In resource-limited settings, the demand for quality data and the interest in health information systems has increased due to the need for country-level progress reports towards attainment of the United Nations Millennium Development Goals [4, 5], and due to global health initiative funding, such as the United States President's Emergency Plan for AIDS Relief (PEPFAR); the Bill and Melinda Gates Foundation; and the Global Fund to Fight AIDS, Tuberculosis, and Malaria.

Many low- and middle-income countries experience challenges related to producing quality health data [6, 7]. Contributing factors include weak linkages between M&E systems and data generation points [8, 9], and inadequate harmonization of multiple data collection and reporting systems [8, 10, 11]. Additional challenges include poorly coordinated M&E systems characterised by duplication of data collection and underutilization of nationally-defined tools [12–15]. A frequently documented challenge related to producing quality health data in low- and middle-income countries is the shortage of trained human resources [8, 16–20].

To improve data quality in resource-limited settings, it is important to address the shortage of trained human resources. One approach that has been effectively used to address skilled health workforce shortages is task-shifting [21]. This involves the rational redistribution of tasks among workforce teams and includes substituting tasks, delegating tasks, creating a new cadre, or a combination of these [21]. While multiple studies have shown that task-shifting can be an effective strategy for strengthening the provision of health services [22–27], there is little documentation in the literature related to task-shifting as a strategy for improving health information systems and data quality [28].

To address data quality and health information challenges in Botswana, multiple donors and stakeholders partnered with the government to implement a task-shifting initiative that established a new cadre of health worker: the district M&E officer. As described elsewhere in literature [28], university graduates in the field of social sciences, with no prior health information exposure, were recruited for the cadre. A total of 51 were initially employed, with most of the districts in the country receiving two officers. They were provided with on-the-job training and mentoring to equip them with the knowledge and skills necessary to carry out M&E responsibilities in health districts across the country. The goal was that the district M&E officers would assume data-related duties that had previously been secondary responsibilities of other health workers, such as nurses. These health workers had been challenged in fulfilling M&E responsibilities because of the increasing workload associated with health program expansion as well as limited training in M&E. The duties of the M&E officer cadre were to strengthen data collection, ensure regular and timely reporting and feedback, promote a culture of data utilization and evidence-based planning, and build M&E capacity within the health system. Approximately three years after the district M&E officer cadre had been established, a qualitative assessment was conducted as part of a larger evaluation of the cadre. The objectives of this assessment were to describe the achievements of the district M&E officer and document lessons learned during the establishment and implementation of this cadre into the existing public health services infrastructure.

## Methods

This qualitative assessment included in-depth interviews with individuals responsible for the development and implementation of the district M&E officer cadre, as well as focus group discussions with the district M&E officers and cadres working closely with them at the district level. The assessment was conducted by the International Training and Education Center for Health (I-TECH) in Botswana, a collaboration between the University of Washington and University of California, San Francisco. A technical working group led by the Ministry of Local Government (MLG) and comprised of multiple stakeholders oversaw the M&E officer evaluation and guided the qualitative assessment. It was approved by research ethics review boards at the Botswana Ministry of Health, the University of Washington, and the United States Centers for Disease Control and Prevention.

The in-depth interviews, administered by trained interviewers, were semi-structured with open-ended questions. They were conducted to provide information on the development of the cadre, achievements resulting from the cadre, and challenges faced during implementation. A total of 18 potential interviewees were purposively sampled through identification by the M&E technical working group, having substantially contributed to the development of the cadre through training, mentoring and supportive supervision. They represented all government institutions, donor agencies, and technical organizations involved in development of the cadre. All the 18 potential interviewees were invited to participate, but 12 were available and interviewed (response rate = 67%); eight from government entities, three from technical partners and one from a donor agency.

Six focus group discussions (FGDs) were conducted in the two main urban districts of the country. The eleven districts within an 80 kilometre radius of the two urban districts were invited to participate. Of these 11 districts, 10 participated in the FGDs, representing 33% of the 29 districts in the country. This included six rural and four urban districts. The FGDs were conducted with three separate cadres purposively selected from the participating districts. This included 12 district M&E officers, 15 district program officers coordinating health programs, and 12 district health managers in charge of health services. Program officers were included because they are beneficiaries of M&E support and some of their data-related duties had been task-shifted to the district M&E officers. A community health nurse and a PMTCT coordinator were invited per district in this group. District managers were included as the supervisors of the district M&E officers, and a District Health Management Team coordinator and District AIDS coordinator or their representatives respectively were invited per district. Additionally, all M&E officers were invited with some districts having two while others had one. On average, there were seven participants in each focus group (range: five to nine). All of the focus group discussions centered on achievements, challenges, and possible solutions to improve the cadre and its work.

With permission from participants, interviews and focus group discussions were recorded and transcribed. ATLAS.ti (Version 6.0, Scientific Software Development) was used as a framework for analysis of the transcripts. A general inductive approach was taken for analyzing the qualitative evaluation data from the in-depth interviews and focus groups [29]. This involved the manual coding of textual data and identification of common themes, in order to condense the data into a summary format and establish links with the evaluation objectives.

## Results

### Achievements

Data from the in-depth interviews and focus group discussions demonstrate several achievements from the establishment of the district M&E officer cadre. These include improved health worker capacity to monitor and evaluate programs within the districts; improved data quality, management, and reporting; increased use of health data for disease surveillance and public health services planning purposes; introduction of district-led operational research activities; and increased availability of time for nurses and other health workers to concentrate on core clinical duties.

#### Improved district-level capacity to monitor and evaluate programs

District M&E officers helped build M&E capacity in the districts by providing in-service training and mentoring to health workers based at the district health offices, health facilities, and civil society organizations. Capacity-building efforts focused on the significance of M&E, data quality (timeliness, completeness, reliability, validity, and accuracy), and data analysis. Health workers received training on the existing data collection tools and data management as indicated by what this district M&E officer said during a focus group discussion:

*“We [the M&E officers] have trained them [health workers] on the importance of data management, and they have started appreciating M&E…Data quality has improved a lot.”*

Training and mentoring in M&E was conducted on-the-job or during scheduled district meetings. Data audit activities and facility support visits were used to identify capacity development needs for health workers, such as basic computer skills. A notable outcome of the capacity-building efforts has been the increased awareness and appreciation of M&E, and the creation of a culture where its recognition has led to its inclusion in district meetings. Two key informants during in-depth interviews respectively said:

*“We also saw a lot of engagement with the districts now. People are beginning to ask a lot of questions regarding the science behind some issues that we included in the data collection tools… this is consciousness. People are beginning to question the inclusion of certain variables in those tools.”*

*“…If you could count the number of times you hear the word “M&E” in workshops nowadays, it is probably 10–20 times more than it was before they [the district M&E officers] came in. Now that is what I’m calling the development of an M&E culture in this country. That, if nothing else, is the ultimate achievement.”*

#### Improved data quality, management, and reporting

Each cadre of interviewee, district M&E officers, program officers, and district managers, acknowledged the contribution of district M&E officers in helping improve data quality, management, and reporting. Improvements in data quality included accuracy, timeliness, validity, and completeness. Introduction of data audits and standardization of data collection tools contributed to the perceived improvements in accuracy and completeness of reporting. The development of tools to track the submission of reports from facilities and feedback given to facilities helped improve timeliness and reporting. Three district managers respectively remarked in focus group discussions:

*“There has been some improvement in terms of timely submissions and completeness [of data and reports], though not a hundred percent."*

*“There has been reasonable improvement in the way the data is collected, [and] the way the data is entered, interpreted, and reports made.”*

*“Even as management, we now get proper and true representation of the [Health] data from the facilities. Now, we are confident of the validity of the data.”*

District M&E officers also introduced systematic filing of health reports by maintaining centrally placed manual files and creating electronic databases, thereby improving data management. This improved both the availability and access to aggregate health data. In focus group discussions, a program officer and a district M&E officer, respectively, reported:

*“Their [M&E officers’] data is safely kept in a way that if anyone comes into the office and is enquiring about something, it does not take long to find information…”*

*“At my district, I met with program officers to train them on making electronic files and saving data on them. Initially they were just recording manually, and you would hardly find a trace of that information later on when you wanted to make reference to it. Now, we keep both manual and electronic folders, and accessibility to data has improved.”*

#### Increased use of health data for disease surveillance, planning and project management

The presence of the district M&E officers improved the use of local data for disease surveillance purposes. Data were analyzed, and disease trends were reported to the district health management teams. District M&E officers became important members of disease control teams and contributed to outbreak investigations. During a focus group discussion, a district manager expressed the following:

*“They [M&E officers] are able to alert the head professionals when the statistics are high, especially the emergency of outbreaks like the diarrhoea outbreaks.”*

In addition to surveillance, improved data quality better informed evidence-based planning at the district level. Health program monitoring and evaluation data has been used to guide the planning process and determine priorities. Two district managers remarked during a focus group:

*“…even though we were using data for evidence-based planning, there wasn’t quality data that really informed us appropriately. But now, the district M&E officers… really work on this data and ensure that it is quality data that we need for planning. This is a priority in the district now, you are basing [decisions] on reliable data, unlike in the past we were using data which is not reliable.”*

*“When you put a priority, as part of plan, they will say that the evidence does not reflect that. You can’t put that as a priority because your numbers for this [are] not indicative.”*

The district M&E officers were reported to monitor implementation of district health plans and provide district health teams feedback on progress towards objectives. This strengthened project management, and districts were able to keep track of implementation of prioritised activities. A district manager expressed the following:

*“They [district M&E officers] crosscheck to see if we are on track, if we have attained our goals, if we are following the set objectives and timelines, and if the funds are directed towards the planned activities or diverted to other ‘emerging needs’, which are not a priority in the district.”*

#### Introduction of district-led operational research activities

District M&E officers introduced operational research in the districts they supported in order to better understand health events. Operational research questions were generated from routinely collected data. Findings from research were shared with the district teams, and some, at a national HIV/AIDS research conference. A program manager and a key informant, respectively, stated:

*“…I understand the district M&E officer did a needs assessment to try to address the issue of teenage pregnancy in district[X]. That needs assessment was used to inform the evidence-based plan.”*

*“..they started getting involved in conducting basic research and sharing their results at conferences, the first one being a National HIV/AIDS Research Conference where they presented some research activities that they were doing.”*

#### Increased availability of time for nurses and other health workers to concentrate on core clinical duties

The district M&E officers took on M&E responsibilities that were previously conducted by nurses and other health workers as secondary activities to core program and clinical responsibilities. This allowed such health workers to concentrate on delivering quality clinical services and focusing on implementation of quality, priority health programs. A district manager reported:

*“So, when the district M&E officers came in, they relieved the community health nurse in such a way that the community health nurse is able to go to facilities to attend to such programs as child health and others. The district M&E officer then took up [data responsibilities] for different HIV programs.”*

Despite taking over M&E responsibilities, district M&E officers worked closely with other health workers coordinating different programs as most program data comes through them.

### Lessons Learnt

Lessons learnt from the qualitative assessment of the M&E officer cadre included the importance of clarifying of roles for newly established cadres, aligning resources and equipment to expectations, and ensuring stakeholder collaboration. Another lesson learnt was the need to ensure retention of new cadres.

#### Clarity regarding how tasks are shifted was essential for the acceptance of a new cadre within the system

When district M&E officers were deployed to the districts, their duties had not been well articulated and communicated to them, their supervisors, nor their colleagues. As a result, they were often tasked with other activities outside M&E. District M&E officers felt that this lack of clarity related to roles and responsibilities was an impediment to developing cooperative working relationships with colleagues. In some instances, district M&E officers were perceived as a threat by program officers to their job security since the cadre had been deployed to the districts to take over some of the duties previously conducted by program officers. A key informant during an in-depth interview and a program officer in a focus group discussion remarked:

*“But at the end of the day, we also needed to have prepared district teams themselves… That part [orientation] was not done, and that was a serious omission....”*

“*The issue is that their role was never clearly defined and therefore we didn’t know how we are to work with them. If their roles were clear to us, we would have come up with a way so that it works easier for everybody since we would be having an understanding.”*

#### Expectations of a newly established cadre must be aligned to available resources

District M&E officers reported that they often struggled to execute their duties effectively due to inadequate resources. These included transport and communication infrastructure challenges, and unreliable access to computers and the Internet. Transport challenges oftentimes affected planned facility-level activities like data audits, while limited access to telephone and facsimile affected reporting and feedback. Unavailability of internet in some districts made it difficult for the district M&E officers to use web-based data reporting tools, such as the district health information system (DHIS). A district M&E officer and a key informant, respectively, reported:

*“I have never used DHIS. They introduced DHIS 2.0, and I am supposed to be connected to the internet, but I don’t have it at my facility. I don’t see it working.”*

*“A lot of things were expected from them [district M&E officers], but without giving enough logistical support. There was no internet in the office. This was challenging for them.”*

#### Planning for career growth and retention is critical

The district M&E officer cadre was initially donor-funded, on fixed-term contracts without a career trajectory, compared to other health workers employed directly through the public health service establishment. While plans were available for a smooth transition of the district M&E officers into the public service structure, being on contract led to job insecurity. As a result, some district M&E officers resigned from their positions. A program officer expressed the following:

*“They are watching their colleagues moving up the ladder! They are just in one place. Even the good ones…. we are going to lose them if the trend continues.”*

Realising the need to retain the district M&E officers, stakeholders agreed to offer an additional allowance as a form of incentive to retain the district M&E officers. Despite this effort by stakeholders, job security still remained a concern. Two key informants remarked:

*“…strategies were put in place to retain them. And hence, there was what we called a “contractual allowance.”*

*“..the stakeholders met regarding retention of the officers and gave them 20% of their salary but this didn’t address the issue of [job]security.”*

#### Stakeholder collaboration and building local capacity contributed to success and sustainability

Multiple stakeholders, including national institutions, donor agencies, and technical organisations, collaborated in the establishment and implementation of the district M&E officer cadre. These stakeholders worked together as members of the technical working group that oversaw the establishment of the cadre, and brought together various areas of expertise that were critical for the cadre. The recruitment of nationals of Botswana for the district M&E officer positions contributed to sustainability of the M&E discipline within Botswana. Despite some of the M&E officers resigning, they joined other organisations within the country as M&E officers and have continued to contribute to the national M&E efforts. Two key informants indicated:

*“And I would say one of the biggest successes of the project, these were all local individuals. They were all hired locally and even if they move, they are still in country, working with other partners and donors, still in the same field. So I would say we did build capacity in the country when it comes to monitoring and evaluation”*

*“… a lot of the district M&E officers now are still in the field of monitoring and evaluation, with different partners, with different United States Government-funded partners and government.”*

Additionally, senior M&E officers at the national level were mentored to ensure sustained capacity to provide technical support to the district M&E officers. For continuous capacity building in M&E, stakeholders also developed self-guided training materials, consisting of workbooks and a classroom-based curriculum. These were meant for ongoing, self-guided use by district M&E officers. Two key informants said:

*“Mentoring was offered to national-level senior M&E officers to ensure that the district M&E officers continue getting the right support.”*

*“Stakeholders developed training materials which should really be utilised; they should be availed so that these officers utilize these materials for their own growth on the job and skills development*.”

## Discussion

This assessment demonstrated that an innovative human resources approach in Botswana contributed to improvements in the quality of health data. District M&E officers improved M&E capacity through training and mentoring of health workers employed in the public sector and at civil society organisations. An increased awareness and appreciation of M&E helped create a culture where M&E was included in meetings, trainings, and workshops. The district M&E officers played a key role in the introduction of data audits at public health facilities, which led to improvements in data quality and increased its utility for disease surveillance, evidence-based planning, and project management at the district-level. Additionally, the district M&E officers also introduced operational research activities to address research questions and health issues identified through routine data collection in the public health system.

This assessment highlights the importance of human resources in relation to ensuring data quality. Several other studies also indicated that data quality improved by addressing human resource capacity issues, mainly through training of health workers on the importance of public health information and regular feedback [6, 18, 30, 31]. In Malawi and South Africa, it was found that data quality improved through provision of supportive supervision [19, 32]; implementation of regular audits [19]; and the introduction of operational research [33]. Other initiatives, such as the transition from paper-based to electronic records, have also been shown to improve data quality [34]. This assessment has shown that many of these data quality improvement activities can be conducted by establishing a dedicated cadre for monitoring and evaluation.

A key lesson learnt from this assessment was the need for adequate preparation for successful implementation of a task-shifting initiative. This includes clarification of roles to those working with and supervising the cadre, and the provision of adequate resources for use by the new cadre in discharging its duties. Lack of role clarity was shown to lead to reluctance to change in the implementation of a task-shifting initiative in Uganda [35], making it difficult for the new cadre to perform its duties. Shortage of resources, including for transport were reported elsewhere during site visits targeting the same cadres [28]. The district M&E officer cadre was employed on short-term contracts, which created job insecurity as long term funding plans were not yet in place, a key requirement for successful task-shifting initiatives [36]. In Tanzania, lack of long-term funding plans were found to have negative implications for promotion and career development when implementing task-shifting approaches [37], an issue that was found in this assessment.

The establishment of the cadre was a country-led initiative with collaboration from multiple stakeholders, which is consistent with guiding principles for sustainable national M&E systems [15]. The M&E officers were locally recruited, a human resources approach that is a key aspect for a sustainable information system [17]. The achievement of the Millennium Development Goals has put an emphasis on long-term sustainability of health programs [38]. Plans promoting long term sustainability of the cadre were initiated when the cadre was developed. A few months after the completion of data collection for this assessment, half of the district M&E officers had been absorbed into the public service establishment, demonstrating the possibilities of achieving long term sustainability for donor funded initiatives.

This assessment has some limitations. Key informants from the in-depth interviews may have demonstrated social desirability in their responses, due to their participation in the development of the district M&E officer cadre. Convenience sampling of M&E officers, program managers, and district managers for the focus group discussions from the vicinity of the two urban areas may have resulted in the lack of reporting of certain unique factors affecting the success of district M&E officers in the rural districts. The creation of the district M&E officer cadre happened at a time when the health workforce in Botswana was also gaining exposure to M&E through different training programs. This exposure may have contributed to the improved M&E capacity at district level and the subsequent changes in data quality, management, and use. The lack of quantitative data to provide an objective measure of data quality is yet another limitation to this assessment. As such, it is difficult to attribute the stated successes to the cadre. Use of iterative questioning during data collection and triangulating findings from focus group discussions and in-depth interviews strengthen the validity of the results. However, there is need for further research to assess whether alleviation of such M&E tasks from other health workers results in improved quality of patient-level care and health outcomes.

## Conclusion

This assessment demonstrated that the establishment of a dedicated health information cadre can improve the health information system through capacity-building, data quality and data use for decision-making. The cadre can be established in a sustainable manner if the initiative is country-led, and it is done in collaboration with multiple stakeholders supporting different components of the program. The transition to full country support of such donor-supported initiatives is achievable but requires consecutive years of support with a clearly defined transition plan and government retention strategy.
